# Synthesis and Characterization of a New Aluminosilicate Molecular Sieve from Aluminosilica Perhydrate Hydrogel

**DOI:** 10.3390/ma13235469

**Published:** 2020-11-30

**Authors:** Haiqiang Ma, Kun Jiao, Xiangyu Xu, Jiaqing Song

**Affiliations:** 1 State Key Laboratory of Chemical Resource Engineering, Beijing University of Chemical Technology, Beijing 100029, China; 18101218769@163.com (H.M.); xuxy@mail.buct.edu.cn (X.X.); 2 College of Chemistry and Molecular Engineering, Peking University, Beijing 100871, China; lovelyjjq0809@163.com

**Keywords:** aluminosilica perhydrate hydrogel, molecular sieve, peroxides, silicon-aluminum source, synthesis method

## Abstract

A novel structure aluminosilicate molecular sieve, named BUCT-3, was prepared by dynamic hydrothermal synthesis, and the critical factor to obtain the new structure is using an active silicon and aluminum source, aluminosilica perhydrate hydrogel. Meanwhile, only high content of O-O bonds can ensure the pure phase of BUCT-3. Through the characterization of x-ray powder diffraction (XRD), Fourier transform infrared spectra (FTIR), scanning electron microscopy (SEM), and so on, some structure and morphology information of BUCT-3 molecular sieves as well as the special silicon and aluminum source was obtained. It’s worth noticing that the O-O bonds of reactants can be reserved in the products, and thus, help us to get a new structure with cell parameters a = 8.9645 Å, b = 15.2727 Å, c = 11.3907 Å, α = 90°, β = 93.858°, γ = 90°. The crystal system is monoclinic. Though the thermostability of BUCT-3 is not satisfactory, its potential application derived from O-O bonds cannot be neglected.

## 1. Introduction

Zeolites are microporous crystalline inorganic materials with well-defined pore systems. Since the 1940s, the study of zeolites with new structure has attracted a great deal of interest for their specific catalytic or adsorption properties [1,2]. Much effort has been devoted to developing novel molecular sieves [3,4].

Generally speaking, molecular sieves with new structures can be obtained in three ways: (i) Zeolites synthesized by using structure-tunable organic ammonium salt template: This method usually requires the use of organic amine/ammonium cations as structure directing agents (SDAs). Although designing new structures of templates is expected to be the most useful way to synthesize new structural molecular sieves, it’s also the most complicated method [5,6,7]. (ii) Molecular sieves synthesized by using heteroatom substitution [8]: heteroatom molecular sieves using other elements, such as Ti, Cr, Zr, and Ga [9,10,11], partially substitute silicon, aluminum, or phosphorus in the framework to form heteroatom-containing molecular sieves. These elements that enter the framework can be main group elements, or transition elements from 2+ to 5+. Due to the introduction of specific non-metal or metal atoms, many new molecular sieves with special structures have been successfully synthesized. (iii) Molecular sieves synthesized by topotactic transformation [12,13]: two steps are required to obtain three-dimensional structure molecular sieves from two-dimensional layered precursors: (a) Preparation of layered precursors by traditional hydrothermal synthesis; (b) the two-dimensional precursor undergoes interlayer dehydration and condensation through high-temperature solid-phase reaction to form a three-dimensional molecular sieve, which is the process of topological transformation [5].

Many parameters can also influence the crystallinity, morphology, and even structure of molecular sieves, such as SDA/TO_2_, OH^−^/TO_2_, and H_2_O/TO_2_, as well as the synthesis temperature, crystallization time, and so on [14].

Another important feature of molecular sieves synthesis is that even if the other conditions of crystallization are the same, different framework structures may be obtained if different sources of reactants are used, especially the essential element of structure. It is known that the use of different silica sources may affect the type of zeolites [15,16,17], crystal size [17,18,19,20,21,22,23,24,25], morphology [20,21,25], and the rate of crystallization [15,16,19,20,21,22,23,24], since the fragile silicate intermediates releasing during the process of the silica dissolution plays an important role in the framework formation [26]. A typical example [27] is that scientists from Union Carbide Corporation converted an inert silicon-aluminum source into an active source, and synthesized a series of new molecular sieves ranging from A to Y, indicating that the activity of the source affects the structure of the molecular sieve. This work has inspired us to synthesize new molecular sieves by changing the activity of raw materials.

Hydrogen peroxide is a clean and green chemical product. Its special electronic structure enables it to increase the activity of some reactions. Compared with the design of complex, expensive, and polluting organic templates to synthesize new molecular sieves, relatively inexpensive hydrogen peroxide is used to treat the reaction raw materials in order to change the activity of the source, so as to carry out the reaction process with low energy consumption. Considering cost-effectiveness and environmental cleanliness, it is advantageous to change the synthesis method of raw material activity to obtain new molecular sieves.

In this article, a new kind of aluminosilicate molecular sieve with novel structure was obtained by using a usual template, tetrapropylammonium hydroxide (TPAOH) as the SDA, and unusual reactants, aluminosilica perhydrate hydrogel as the silicon and aluminum source. This is an unstable structure with peroxide bond that makes the structure elucidation very difficult. However, a combination of x-ray powder diffraction (XRD) and Fourier transform infrared spectra (FTIR) gives some partial structure of the novel material. We also explored in detail the effects of different silicon and aluminum sources, temperatures, and time on the products.

## 2. Materials and Methods

### 2.1. Chemicals

The chemicals used in our study were as following: tetraethoxysilane (TEOS, 28 wt% SiO_2_, Guang Dong Fine Chemicals Co., Ltd., Guangdong, China), tetrapropylammonium hydroxide (TPAOH 25 wt% in water, Shanghai Aladdin Bio-Chem Technology Co., LTD, Shanghai, China), Ammonia solution (NH_3_ 25–28 wt%, Beijing Chemical works Co., Ltd., Beijing, China), hydrogen peroxide (H_2_O_2_ 30 wt%, Beijing Chemical works Co., Ltd., Beijing, China) and deionized water, sodium aluminate (Al_2_O_3_ 50–56 wt%, Na_2_O 40–45 wt%, Fisher, UK), and sodium silicate (Na_2_SiO_3_, Guang Dong Fine Chemicals Co., Ltd., Guangdong, China).

### 2.2. Synthesis

Silica gel: A certain amount of ammonium fluoride was dissolved in deionized water according to the molar ratio of 0.004:4 to obtain solution A. Then, solution A was added dropwise to Solution B consisting of ethanol, water, and TEOS in a molar ratio of 8:4:1. After stirring until uniform, a transparent gel is obtained, which is dried in an oven at 50 °C for 1 d, and then roughly ground after taking it out. Finally, it is calcined in a muffle furnace at 800 °C for 2 h.

Aluminosilica perhydrate hydrogel-1 (SiAl-1): Aluminosilica perhydrate hydrogel-1 was prepared by dissolving a fixed amount of NaAlO_2_ and Na_2_SiO_3_ (Si/Al = 25) in deionized water, and then H_2_O_2_ were added dropwise to the above solution and stirred for 1 h at room temperature. After drying at 50 °C for 24 h, the product was named as SiAl-1. The initial feed ratio of the gel was: Al_2_O_3_:SiO_2_:H_2_O_2_:H_2_O = 1:50:194:2162.

Aluminosilica perhydrate hydrogel-2 (SiAl-2): TEOS was dissolved in the mixture of ethanol and deionized water, followed by a small amount of ammonia solution to promote the hydrolysis process. Subsequently, NaAlO_2_ solution was added to the silica gel with fixed Si/Al ratio 25. After stirring for a few minutes, H_2_O_2_ was then added dropwise to the mixture with continuous stirring at room temperature for 0.5 h. After drying at 50 °C for 24 h, the product was named as SiAl-2. The initial feed ratio of the gel was: Al_2_O_3_:SiO_2_:H_2_O_2_:H_2_O = 1:50:365:1720.

Preparation of aluminosilicate molecular sieves: All samples were prepared by dynamic hydrothermal synthesis. The mixture of TPAOH, silica source, aluminum source, and deionized water was directly transferred into a teflon autoclave (25 mL capacity) with continuous stirring for at least 6 h. Finally, the autoclave was transferred to a homogeneous reactor at 70–120 °C for 2–4 days. Then, solid products were centrifuged, followed by repeatedly washing with deionized water and drying at 60 °C overnight. The initial feed ratio of the mixture was Al_2_O_3_ SiO_2_:TPAOH:H_2_O = 1:50:25:2500.

### 2.3. Characterizations

The X-ray diffraction (XRD) measurement was performed to obtain the phase information of the molecular sieve samples. The XRD data were collected by UItima III diffractometer (Rigaku Corporation, Tokyo, Japan) with Cu Kα radiation, the scanning range is from 5° to 50°, scanning 3 times at a rate of 10° min^−1^.

Scanning electron microscopy (SEM) images were performed using ZEISS SUPER55 scanning electron microscope (ZEISS, Jena, Germany). Its acceleration voltage is 3 kV, and the magnification is 1 × 10^3^–1 × 10^6^.

Infrared spectra (IR) were collected on a Vector 22 FTIR spectrometer (Bruker, Leipzig, Germany) to obtain some structural information of the new molecular sieve (400–5000 cm^−1^).

In-situ variable temperature X-ray diffraction data were collected on a modified Bruker D8 Advance diffractometer equipped with MRI high temperature attachment, a graphite monochromator and LynxEye detector (Bruker, Leipzig, Germany), using a Cu Ka radiation source (¼ 1.5418 Å) in Bragg-Brentano geometry, with a step width of 0.02° in the 2θ range from 5° to 50°, and the keeping time was 2 s per step. The heating rate was 10 °C min^−1^, and the sample was equilibrated for 300 s prior to XRD data collection at a certain temperature. 

Element analysis of products were characterized by elemental analysis (EA) using a Vario El elemental analyzer (elementar Analysensyteme GmbH, hanau, Germany).

X-ray photoelectron spectroscopy (XPS) measurements conducted by AXIS Ultra DLD (Kratos, Manchester, UK) with Al Kα radiation (149.94 W).

## 3. Results and Discussion

As mentioned in the introduction, different silica or aluminum sources strongly influence the nucleation and crystallization of zeolites. Our initial experimental idea was to study the effect on ZSM-5 by using different silicon and aluminum sources. Yet, considerable work has examined the influence of common silicon sources, like tetraethylorthosilicate (TEOS), fumed silica, and colloidal silica, sodium metasilicate [28]. For this reason, we carefully selected a special silicon source and aluminum source, aluminosilica perhydrate hydrogel, to investigate the influence on the structure of ZSM-5. At the same time, by adjusting the synthesis conditions, we hope to synthesize a molecular sieve with a new structure.

### 3.1. Characterization of Raw Materials

**Figure 1:**
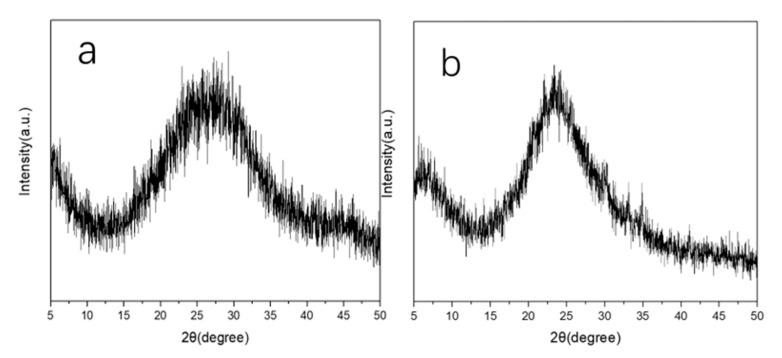
X-ray powder diffraction (XRD) patterns of sample (**a**) SiAl-1 and (**b**) SiAl-2.

**Figure 2:**
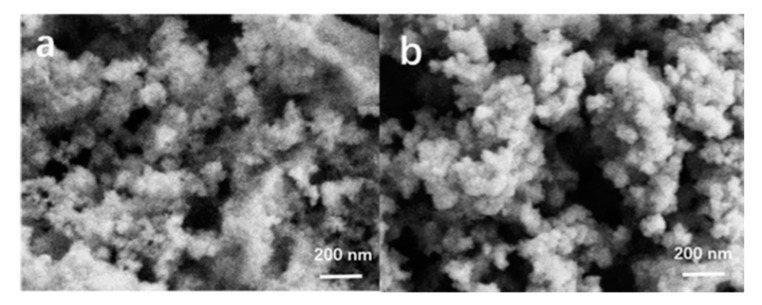
Scanning electron microscopy (SEM) images of sample (**a**) SiAl-1 and (**b**) SiAl-2.

Prior studies [29,30] have reported methods for preparing silica-alumina gel but there are few reports on the preparation of aluminosilica perhydrate hydrogel. In fact, the preparation method of the aluminosilica perhydrate hydrogel is similar with the silica-alumina gel, except that part of the water in the silica-alumina gel was replaced by hydrogen peroxide solution when the aluminosilica perhydrate hydrogel was prepared. Peralumina or persilica is a complexation of alumina and hydrogen-peroxide, which contain peroxide or hydroperoxide groups. In our experiment, two kinds of aluminosilica perhydrate hydrogel were obtained, namely SiAl-1 and SiAl-2, which differed in the silicon and aluminum source used in the process. Figure 1 shows the XRD of SiAl-1 and SiAl-2; the bulging peaks show a clear amorphous phase. Figure 2 shows SEM images of the silicon and aluminum source SiAl-1 and SiAl-2. The images of both materials demonstrate spherically shaped nanoparticles of 20–50 nm. Obviously, the sample of SiAl-2 is more uniform and dispersal. Owing to the hydrolysis rate of TEOS in water is slow, the reaction process is easy to control, and the prepared colloidal particles have the same size without agglomeration.

**Figure 3:**
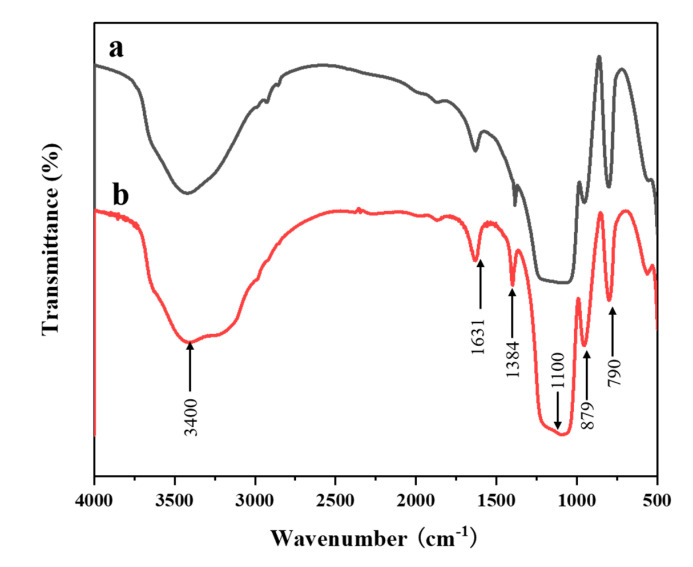
Fourier transform infrared spectra (FTIR) spectra of sample (**a**) SiAl-1 and (**b**) SiAl-2.

The FTIR spectroscopy is an effective method for studying hydrogen peroxide complexes. Due to the unique electronic structure and geometric configuration of the hydrogen peroxide molecule, it can form a strong hydrogen bond with the hydroxyl group on the surface of the silica gel [31]. To confirm the O-O bonds were actually formed in the prepared aluminosilica perhydrate hydrogel, SiAl-1 and SiAl-2 sample was studied by the FTIR technique. Figure 3 shows the FTIR spectra of SiAl-1 and SiAl-2. The hydrogen bond formed by the O-H groups (Si-OH) with H_2_O and H_2_O_2_ molecules was found to have a strong absorption peak near 3500 cm^−1^. However, the bending vibration of H-O-O (H_2_O_2_) connected to the silica framework was found to be weakly absorbed at 1300 cm^−1^. Previous studies [32,33] show that the sharp peak at 876 cm^−1^ should be assigned to (O-O) stretching vibration of silica perhydrate, relatively speaking, the alumina perhydrate spectrum shows a broad peak here. Moreover, compared with SiAl-1, SiAl-2 has a relatively sharper peak, which means the concentration of O-O bond of SiAl-2 is higher than that of SiAl-1, since the sample/KBr ratio was fixed in all the FTIR analysis.

**Figure 4:**
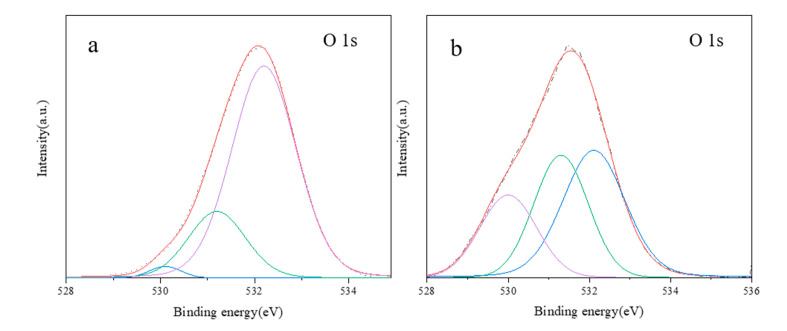
X-ray photoelectron spectroscopy (XPS) of sample (**a**) SiAl-1 and (**b**) SiAl-2.

In order to study the species and relative content of oxygen in aluminosilica perhydrate hydrogel, high-resolution XPS spectra of O were used for peak fitting. Figure 4 shows XPS of sample (a) SiAl-1 and (b) SiAl-2. As can be seen from the figure, the spectra of SiAl-1 and SIAl-2 can be fitted into three components with peak positions of 530.1, 531.2, and 532.2 eV. According to previous research reports [34,35], the peak position of O_2_^2−^ 1s was found at 530 eV. The energy peak positions at 531 and 532 eV should correspond to the surface OH- and bulk T-O-T (T = Si, Al). At the same time, we have summarized the O 1s content of different species in SiAl-1 and SiAl-2 according to the quantitative calculation method of XPS, as shown in Table 1. It can be clearly seen from the table that the O_2_^2−^ content in SiAl-2 is much higher than SiAl-2. This may be due to the preparation method of SiAl-2, that is, the continuous dropwise addition of hydrogen peroxide during the TEOS hydrolysis process can firmly lock the peroxide bond. This is completely consistent with the results of infrared analysis. Consequently, we successfully incorporated the O-O bond into the aluminosilica gel.

### 3.2. Optimized Synthesis and Characterization

ZSM-5 is a zeolite with MFI framework structure, usually prepared by hydrothermal synthesis, the reaction conditions were as follows: Al_2_O_3_:SiO_2_:TPAOH:H_2_O = 1:10–60:10–50:200–3000 at 100–175 °C for 1–8 days [36,37]. In our experiments, tetrapropylammonium hydroxide was chosen as the SDA. Two highly active aluminosilica perhydrate hydrogel were selected as the source. By contrast, silica gel and NaAlO_2_ were selected as the common source of silica-aluminum. Since the silicon and aluminum source have peroxide groups, which have relatively high reactivity, the reaction should better be performed at a low temperature, like 90 °C. In order to intuitively express the synthesis conditions and results, we list the source, temperature, time, and phase in Table 2.

**Figure 5:**
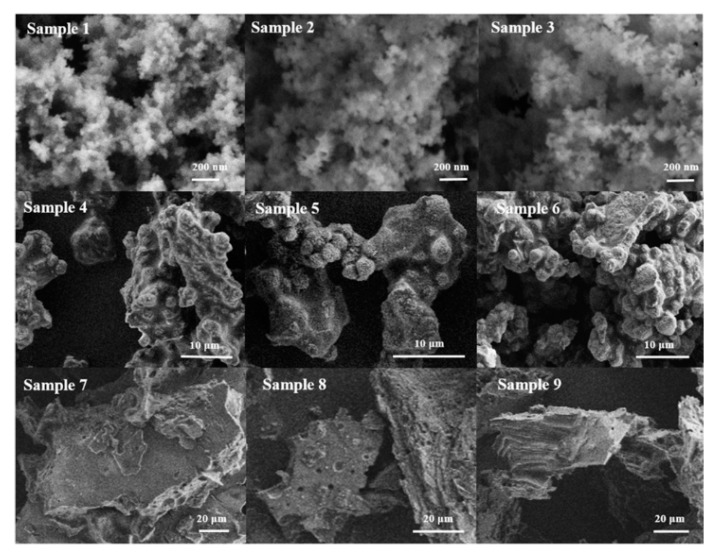
SEM images of Sample 1–9 with different reaction conditions.

The SEM images of Sample 1–9 are shown in Figure 5. Notably, the morphologies of products obtained by using different silicon and aluminum sources are different. Thereinto, Samples 1–3 are nanosized particles, while Samples 4–6 are a mixture with two morphologies, including the spherical morphology and plate-like morphology. As for Samples 7–9, a plate-like morphology with lamellar structure is formed.

**Figure 6:**
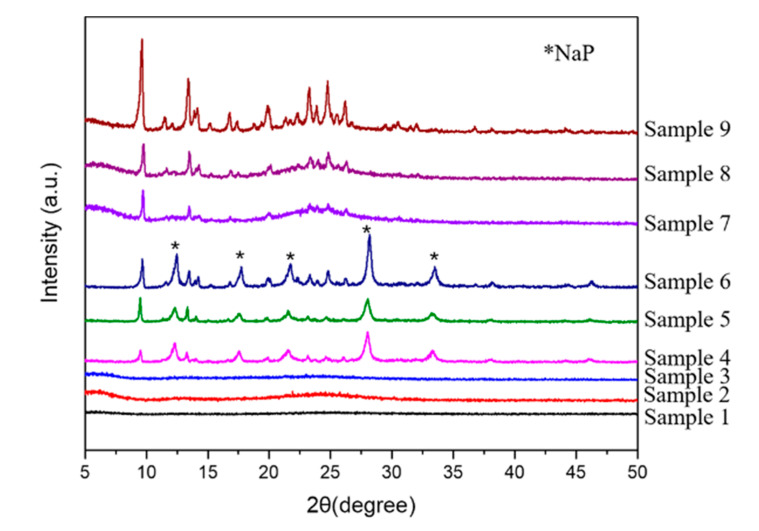
XRD patterns of Sample 1–9.

It can be seen from Figure 6 that except for the samples prepared with SiO_2_ and NaAlO_2_ as raw materials (Sample 1, 2, and 3) are amorphous, other samples have obvious characteristic peaks of BUCT-3 molecular sieve at 2θ = 9.68, 13.4, and 24.78°, whereas, the samples (Sample 4, 5, and 6) with SiAl-1 as source have characteristic peaks of NaP zeolite at 2θ = 12.51, 17.74, 21.71, 28.2, and 33.5°. However, no impurity peak (Sample 7, 8, and 9) was present in the XRD patterns when SiAl-2 was chosen as the starting source. Meanwhile, the crystallinity from Sample 7 to Sample 9 increased with increase of reacting time and temperature, which can be clearly seen from the height of peak at 2θ = 9.68°.

As shown in Table 2, there are three kinds of phases in the products. In addition to the amorphous form, there are a known phase NaP zeolite, and a new phase of unknown structure, named BUCT-3. It’s worth noticing that this new kind of molecular sieve can always be synthesized when the silicon and aluminum sources were treated with H_2_O_2_ under all conditions. On the contrary, when common silicon and aluminum sources, SiO_2_ and NaAlO_2_, were used in these conditions, the products were always amorphous. This implies that at this temperature, due to the low activity of ordinary source, the nucleation and growth of crystals are slower, and the final appearance is amorphous. Overall, these results are in excellent agreement with the SEM images observed. Undeniably, SiAl-1 and SiAl-2 with O-O bond formed by the addition of H_2_O_2_ into the silica-alumina gel have higher reactivity. That is, the O-O bond plays a crucial role in the formation of the new molecular sieves.

**Figure 7:**
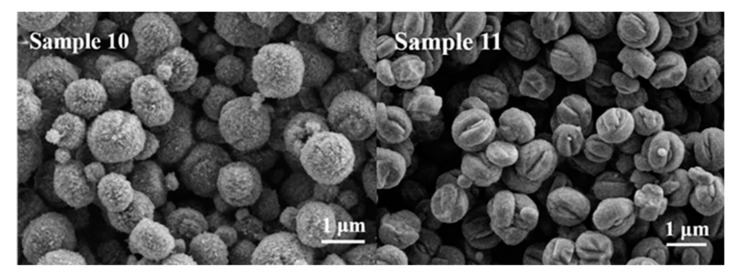
SEM images of Samples 10 and 11.

**Figure 8:**
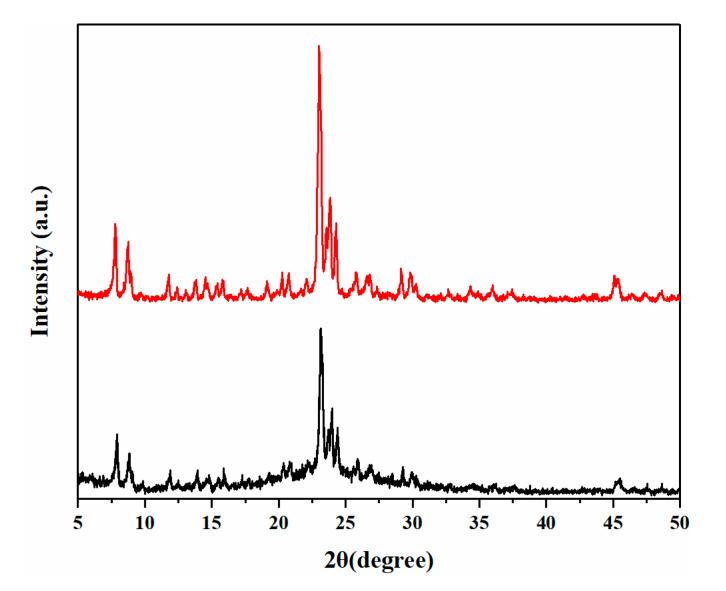
The XRD patterns of Samples 10 and 11.

It is well known that apart from crystallinity and morphology, the crystal phase is also affected by temperature. In order to further increase the crystallinity of BUCT-3, the temperature was raised to 120 °C; the phase composition is shown in Table 3. Figure 7 shows the SEM images of Sample 10 and 11 prepared with different silica-alumina sources at 120 °C. It can be seen that both samples are sphere morphology packed with small particles, and the whole crystal size is about 1 μm. Figure 8 shows the XRD patterns of both samples and they have typical MFI structure. It is clearly seen that further increase in temperature led to the appearance of ZSM-5 zeolites, which is contrary to our expectation. This is because that the O-O bond of SiAl-2 may break at the temperature 120 °C, and therefore, no O-O bond was involved in the reaction, which eventually led to the presence of ZSM-5. This result proves once again that the silica-alumina with O-O bond plays an important role in the formation of the structure of BUCT-3.

### 3.3. Structural Analysis of BUCT-3 Molecular Sieves

Based on the discussion above, when SiAl-2 was chosen as the silicon and aluminum source and the reacting condition was set as 90 °C for 4 days, the product has the highest crystallinity. Therefore, Sample 9 was used to solve the structure of BUCT-3 molecular sieves.

The indexing of Powder X-ray Diffraction data of BUCT-3 was carried out by Fullprof program and the cell parameters are listed in Table 4. Since the result cannot match any known structure in the International Zeolite Association (IZA) database [38], that is, no structures’ cell parameters have the same or multiple relationship with BUCT-3’s, we can conclude that this is a new structure. Since the crystal size of the material cannot meet the conditions of single crystal diffraction, the crystal structure cannot be obtained from single crystal diffraction. Therefore, we consider using the selected area electron diffraction (SAED) method to analyze the sample, but the material is extremely intolerant to the electron beam and begins to melt within 2 min of irradiation (see Figure S1). The instability of materials brings many difficulties to the structural analysis.

**Figure 9:**
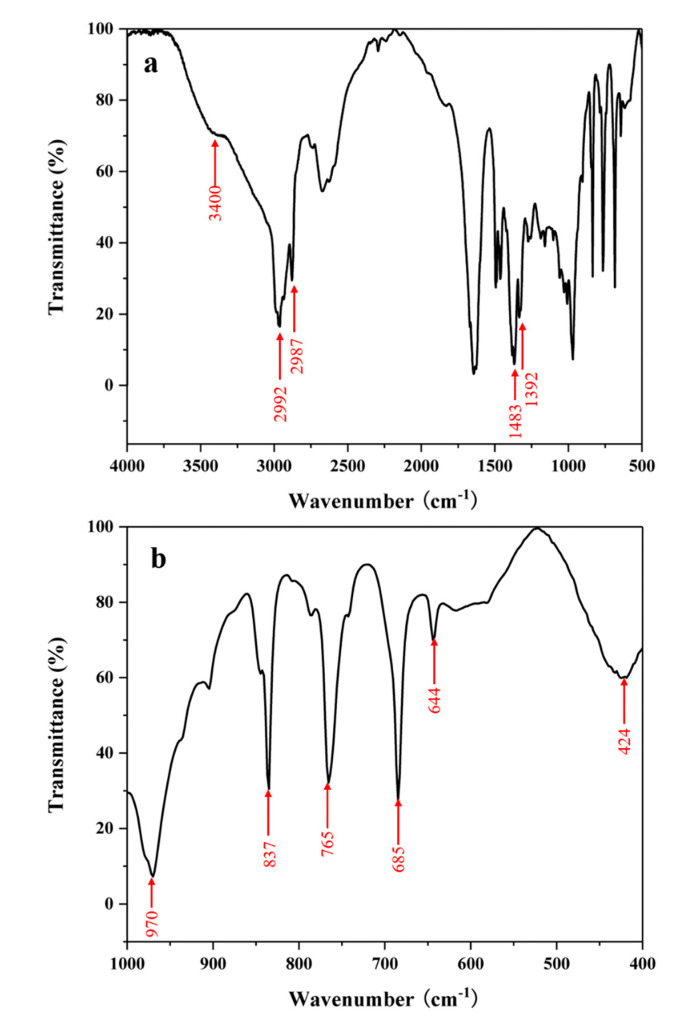
FTIR spectrum of BUCT-3: (**a**) Full FTIR spectrum (**b**) fingerprint region spectrum.

Although the specific structure model of the BUCT-3 is not available, some structural information of the molecular sieve can still be obtained through FTIR, as shown in Figure 9. Based on the detailed study of the infrared bands of zeolite by the KSF [39], some characteristic infrared bands of silica-alumina molecular sieves were identified. The stretching vibration and bending modes of T-O-T (T = Si or Al) can be found to have strong absorption peaks at 424 cm^−1^ and 970 cm^−1^, respectively. The symmetrical stretching vibration peak of the silicon (aluminum) oxygen tetrahedron inside the framework can be found at 650–750 cm^−1^. Furthermore, there are typical stretching vibration bands of the SDA cation, tetrapropylammonium. The peaks near 2992 and 2987 cm^−1^ are characteristic of C-H stretching vibration and the peaks near 1483 and 1392 cm^−1^ are characteristic of N-C unsymmetrical bending vibration. Combined with the result of element analysis in Table 5, it is believed that the SDA cation was completely reserved in the framework, which means the structure directing property of TPA+ was not destroyed by the O-O bonds of the reactants. As mentioned before, O-O stretching vibration can be found at the peak near 873 cm^−1^, and this can explain the poor thermostability of BUCT-3 molecular sieves. On the other hand, the existence of O-O bonds in the framework may help to form a new structure of molecular sieves and extend the application of molecular sieves as well.

**Figure 10:**
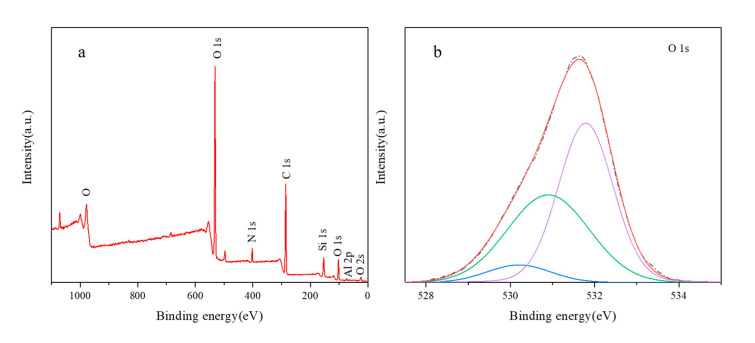
XPS spectra of BUCT-3: (**a**) the full XPS survey spectra, (**b**) high-resolution XPS spectra of O.

The full spectrum XPS of BUCT-3 and the high-resolution XPS of O are shown in Figure 10. From the full spectrum, we can see the main elements such as Si, Al, O, C, and N in the template. From the peak fitting spectrum of O, it can be known that in addition to the skeleton O with binding energy at 532 eV and the surface silanol OH^−^ at 531 eV, there is a most typical O_2_^2−^ peak at 530 eV. Through quantitative analysis and calculation, we have obtained 6.4% O_2_^2−^ of the total O 1s. Since BUCT-3 is prepared from SiAl-2, only a small part of the 23% oxygen in SiAl-2 enters the final structure. This is because most of the O-O is decomposed by heating during the crystallization process. Combined with the above infrared analysis, the conclusion that the peroxide bonds in the aluminosilica perhydrate hydrogel are retained in the framework of the product has been strongly proved.

**Figure 11:**
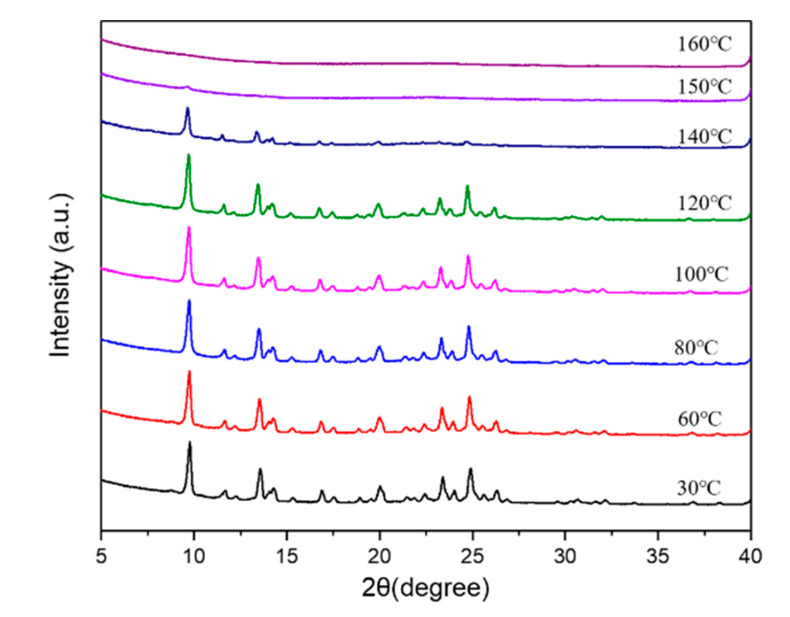
In situ variable temperature XRD patterns of BUCT-3.

In situ variable temperature XRD analysis reveals the thermostability of the as-synthesized BUCT-3 (Figure 11). BUCT-3 molecular sieves started to collapse at 140 °C, which can be clearly seen from the significant decrease of the peaks’ intensities. It is probably because the O-O bonds in the framework of BUCT-3 broke during the heating process. Hence, the optimal calcination condition for BUCT-3 to remove templates is at a lower temperature, like 120 °C, in the presence of ozone and this work is still in progress.

Based on the above conclusions and the hydrogen-bond induced crystallization mechanism of zeolites [40], we can reasonably speculate on the crystallization process of zeolites with the new structure. In the early stage of the reaction, aluminosilica perhydrate hydrogel first depolymerizes into a large number of monomeric and dimeric species with hydrogen-bond (H-O-O). Then, these species condense around TPA+ through the hydrogen bonds on its surface. These hydrogen bonds from the [(HO)_3_SiOSi(OH)_3_]-H_2_O_2_ [31] or SiO^−…^HO-Si [40] serve as the connectors to bridge the silicates together and fuse themselves to form the long-range periodic framework. During the condensation process, the peroxide bond (O-O) of hydrogen peroxide is finally incorporated into the framework.

## 4. Conclusions

In this paper, two kinds of new silicon and aluminum sources obtained by gel-sol method are aluminosilica perhydrate hydrogels and both of them have amorphous phase with nano-sphere morphology. FTIR analysis was used to confirm the existence of O-O bonds in the silicon and aluminum source, SiAl-1 and SiAl-2. Pure phase molecular sieves, BUCT-3, was hydrothermally synthesized by using TPAOH as SDA under mild reacting condition. The key to synthesize this new aluminosilicate is using aluminosilica perhydrate hydrogel with relatively high O-O bond contents, SiAl-2, as the silicon and aluminum source. The cell parameters of BUCT-3 determined by XRD were a = 8.9645 Å, b = 15.2727 Å, c = 11.3907 Å, α = 90°, β = 93.858°, γ = 90°. Since the O-O bonds remained in the structure of BUCT-3, its thermostability is not good. Despite the advances described in the structure elucidation of new materials, there are occasions where the achievement of the real structure of unstable materials is still a complex problem. Therefore, it is necessary to develop a new characterization technique or preparation procedure to solve this extremely unstable structure, so that the detailed structure information of the molecular sieve with peroxide bond in the framework can be obtained. Nevertheless, BUCT-3 molecular sieves may have potential application as a bleaching and oxidizing agent. Besides, this new synthesis method of H_2_O_2_ treatment provides an effective strategy for the design of new molecular sieves.

## Figures and Tables

**Table 1:** O 1s content of different species in SiAl-1 and SiAl-2 from XPS.

Sample	O_2_^2−^ (%)	OH^−^ (%)	Bulk O (%)
SiAl-1	2.1	21.6	76.1
SiAl-2	23.4	33.2	42.3

**Table 2:** The reaction conditions and the phases of Sample 1–9 (Al_2_O_3_:SiO_2_:TPAOH:H_2_O = 1:50:25:2500, 30 r/min).

Sample Number	Silicon and Aluminum Source	Temperature (°C)	Time (d)	Phase
Sample 1	SiO_2_ + NaAlO_2_	70	4	amorphous
Sample 2	SiO_2_ + NaAlO_2_	90	2	amorphous
Sample 3	SiO_2_ + NaAlO_2_	90	4	amorphous
Sample 4	SiAl-1	70	4	NaP + BUCT-3
Sample 5	SiAl-1	90	2	NaP + BUCT-3
Sample 6	SiAl-1	90	4	NaP + BUCT-3
Sample 7	SiAl-2	70	4	BUCT-3 (L)
Sample 8	SiAl-2	90	2	BUCT-3
Sample 9	SiAl-2	90	4	BUCT-3

L: low crystallinity.

**Table 3:** The reaction conditions and phases of Samples 10 and 11.

Sample Number	Silicon and Aluminum Source	Temperature (°C)	Time(d)	Phase
Sample 10	SiO_2_ + NaAlO_2_	120	2	ZSM-5
Sample 11	SiAl-2	120	2	ZSM-5

**Table 4:** The unit cell parameters and crystal system of BUCT-3 molecular sieves by XRD.

Unit Cell Parameters	BUCT-3
a (Å)	8.9645
Standard deviation of a	0.0034
b (Å)	15.2727
Standard deviation of b	0.0067
c (Å)	11.3907
Standard deviation of c	0.0034
α (°)	90
β (°)	93.858
γ (°)	90
Crystal system	Monoclinic

**Table 5:** Element analysis of BUCT-3.

	Weight Percentage (%)				Atom Ratio
Element	N	C	H	O	C/N
BUCT-3	4.62	50.94	9.88	34.56	12.86

## Supplementary Materials

The following are available online at https://www.mdpi.com/1996-1944/13/23/5469/s1, Figure S1: the selected area electron diffraction (SAED) of BUCT-3: (**a**) Irradiate for 1 minute (**b**) Irradiate for 2 minutes (**c**) Irradiate for 2.5 minutes.
