# Supplementary material for: Synthesis and Characterization of a New Aluminosilicate Molecular Sieve from Aluminosilica Perhydrate Hydrogel
Source: Materials (Basel). 2020 Nov 30;13(23):5469. doi: 10.3390/ma13235469 (PMC7731451; doi:10.3390/ma13235469)
Supplement: Supplementary file 1 [file materials-13-05469-s001.zip › materials-974268-2-SM 2.pdf]

materials

Synthesis and Characterization of a New Aluminosilicate Molecular Sieve from Aluminosilica Perhydrate Hydrogel

Haiqiang Ma [1], Kun Jiao [2], Xiangyu Xu [1] and Jiaqing Song [1,*]

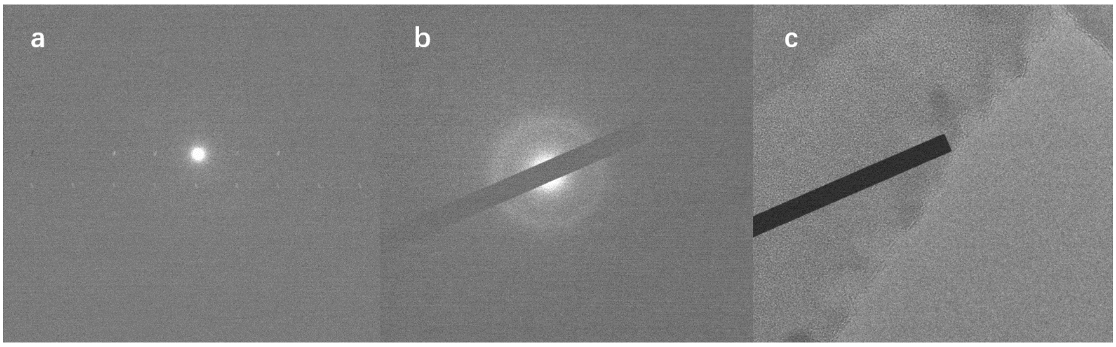

Figure S1. The selected area electron diffraction (SAED) of BUCT-3: (a) Irradiate for 1 minute (b) Irradiate for 2 minutes (c) Irradiate for 2.5 minutes.

It can be seen from the figure that after 2 minutes of focused electron beam irradiation, the material begins to melt, and the crystalline substance becomes an amorphous material with a fuzzy boundary ring. After 2.5 minutes of irradiation, the material melts and the light spot disappears.
